# Tanaidacea of Greece: a preliminary checklist

**DOI:** 10.3897/BDJ.8.e47184

**Published:** 2020-04-22

**Authors:** Panayota Koulouri, Vasilis Gerovasileiou, Nicolas Bailly, Costas Dounas

**Affiliations:** 1 Hellenic Centre for Marine Research (HCMR), Heraklion, Greece Hellenic Centre for Marine Research (HCMR) Heraklion Greece; 2 Hellenic Center for Marine Recearch (HCMR), Heraklion, Greece Hellenic Center for Marine Recearch (HCMR) Heraklion Greece; 3 WorldFish Center, Los Baños, Philippines WorldFish Center Los Baños Philippines

**Keywords:** Tanaidacea, Greece, Aegean Sea, Sea of Crete, Ionian Sea, Eastern Mediterranean, checklist

## Abstract

**Background:**

The checklist of Tanaidacea of Greece was developed in the framework of the LifeWatchGreece Research Infrastructure (ESFRI) project and coordinated by the Hellenic Centre for Marine Research during the period 2013-2015. By applying the Greek Taxon Information System (GTIS) of this project, a complete checklist of species recorded from Greek Seas has been developed. The objectives of the present study were to update and cross-check all tanaidacean species known to occur in Greek Seas. Inaccuracies and omissions according to recent literature and the current taxonomic status were also investigated.

**New information:**

The up-to-date checklist of Tanaidacea of Greece comprises 20 species, classified to 11 genera and five families.

## Introduction

Tanaidacea, comprising an order of Crustacea under the superorder of Peracarida, include species that are amongst the smallest of the benthic macro-invertebrates. Besides their occurrence in deep-sea environments, tanaids are an important component of coastal habitats, such as soft bottoms (Tomassetti and Chimenz Gusso 1998) and vegetated systems (Gambi et al. 1992). They are actually demersal organisms which mainly inhabit the surface layer of the sediments, either in burrows or by constructing tubes or interstitially (Blazewicz-Paszkowycz et al. 2012). Until the 19th century, tanaids had an unclear status, commonly being classified within isopods or amphipods. Until recently, tanaid taxonomy and nomenclature have been undergoing a vigorous revision process (Anderson 2013, Larsen 2002). However, current knowledge of tanaidacean taxa in the world is still significantly under-developed due to lack of competent tanaidacean researchers and taxonomists, appropriate sampling and sample treatment, as well as funding of specific investigations (Blazewicz-Paszkowycz et al. 2012).

Despite several studies from western and central Mediterranean Sea, as well as the coasts of Israel (e.g. Bacescu 1980a, Bacescu 1980b, Bacescu 1981, Guţu 2001, Guţu 2002, Lorenti 2010, Esquete et al. 2012), the tanaid fauna of this region remains understudied in comparison with other macro-invertebrates. The lack of taxonomists within the countries of the Mediterranean region, in combination with inherent identification and sampling difficulties, cannot always be replaced by specialists in other fields and points out the urgent need for efficient solutions in order to correct the present situation (Guţu 2001). As far as Greek Seas are concerned, the most important contribution to their tanaidacean diversity has been made by Stefanidou (1996). Since then, only scattered distribution data on the Greek tanaidaceans have been published (Baxevanis and Chintiroglou 2000, Antoniadou 2004, Chintiroglou et al. 2004, Koulouri et al. 2013).

The first attempt at developing a checklist of Tanaidacea was carried out within the context of the "Greek Biodiversity Database" project (2005-2008, Department of Zoology, Aristotle University of Thessaloniki, Greece). The documented occurrence of these marine species in the Greek Seas was included in a database set up online in 2010. The reference of Koukouras (2010) was specially created by the World Register of Marine Species (WoRMS) / European Register of Marine Species (ERMS, now part of WoRMS), for the list of these marine species provided by the Greek Biodiversity Database during the European project PESI.

The aim of the present study was to provide an updated checklist of Tanaidacea of the Greek Seas. For this purpose, an older list of tanaidacean species was updated according to recent literature and the current taxonomic status of the species.

## Materials and methods

The checklist of Tanaidacea of Greece was developed in the framework of the LifeWatchGreece Research Infrastructure (ESFRI) project and coordinated by the Hellenic Centre for Marine Research during the period 2013-2015 (Arvanitidis et al. 2016). Τhe general principles used for elaborating the preliminary checklist of Tanaidacea of Greece are given in Bailly et al. (2016). The checklist of Tanaidacea was constructed, based on the classification and species records, listed as present in Greece, extracted from the dataset of WoRMS/ERMS for marine species (Koukouras 2010, ERMS 2018). Then, all relevant publications were reviewed and the species reported to date have been added to the list. All taxa were cross-checked and taxonomically updated using WoRMS (WoRMS Editorial Board 2020).

## Checklists

### Checklist of Tanaidacea known to occur in Greek waters

#### Tanaidacea

BEA60D01-9E12-5859-A9C5-EEFCFA945F47

#### Apseudidae

55C86435-1B4D-55EA-BD90-DF460B3F1C15

#### Apseudes africanus

Tattersall, 1925

3B766AE4-4BB3-5C0F-8B6B-FBD3BA117860

#### Apseudes holthuisi

Bacescu, 1961

2E0FFAEF-8884-543A-80DC-950549594A2E

#### Apseudes orientalis

Bacescu, 1961

59284BD5-622F-5E40-8B2D-1AAE30817135

#### Apseudes robustus

Sars, 1882

6CE88DBF-4D85-5CEE-9E1F-170CBC724604

#### Apseudes talpa

(Montagu, 1808)

F6AF75DF-EBD0-5C2C-8381-E8D5E6F8C94D

#### Apseudopsis acutifrons

(Sars, 1882)

3327634E-808A-5CB9-9C70-5F7FE1E8136D

#### Apseudopsis elisae

(Bacescu, 1961)

CE8D6303-D4C8-5C22-83CD-AE93BFA1DF26

#### Apseudopsis latreillii

(Milne Edwards, 1828)

3AF991AF-FFF1-58D8-9640-F6902963ED98

#### Apseudopsis mediterraneus

(Bacescu, 1961)

3F8859D8-CC91-55EB-9CA8-D46CB7A998C0

#### Paradoxapseudes intermedius

(Hansen, 1895)

0EE86616-BA9E-56DA-8405-7F89B776CF08

#### Leptocheliidae

BD3F3783-F32D-58B4-B034-6BD3AA493FB4

#### Chondrochelia savignyi

(Krøyer, 1842)

CE69C8D5-931E-55B0-BE86-D5F1D036D6C2

#### Leptochelia inermis

Dollfus, 1898

F0CE755A-9470-5864-B811-2A684A39D627

#### Leptochelia mergellinae

Smith, 1906

7A898B6C-1920-5ED7-AD25-ABFF592217D1

#### Pseudoleptochelia anomala

(Sars, 1882)

1B1C2D08-CDF7-5767-84E1-BC319D342A51

#### Leptognathiidae

B7776133-FEAD-590B-B4F6-2AF113C67E01

#### Leptognathia breviremis

(Lilljeborg, 1864)

3BDAC777-85FE-5D50-9F1D-98414D09111E

#### Pseudoparatanais batei

(Sars, 1882)

8F7D2E21-E4E6-5CDE-89F4-BEFEC223D3E9

#### Parapseudidae

B1939B9C-0BE3-5415-8EE4-5510C097A763

#### Parapseudes latifrons

(Grube, 1864)

7989BA53-0A7E-5F4B-96E6-572CF1B0B6D8

#### Tanaididae

B4B954CA-1E0E-56EF-84C3-22DB830481A4

#### Hexapleomera robusta

(Moore, 1894)

A6619349-10EF-5DE2-90EA-472D4D2110FC

#### Tanais dulongii

(Audouin, 1826)

69F1244E-D180-574F-BF43-5C5B12502FDE

#### Tanais grimaldii

Dollfus, 1897

34571B34-C0C2-5247-A8DE-CBD593B4BC33

## Discussion

Amongst marine crustaceans, only checklists for Cumacea, Mysida and Lophogastrida and Stomatopoda have been published so far from the Greek Seas (Koulouri et al. 2016a, Koulouri et al. 2016b, Koulouri et al. 2020). A total of 20 species, classified to 11 genera and five families, constitutes the updated checklist of Tanaidacea of Greece. According to Guţu (2001), who has studied the crustacean fauna belonging to the order of Tanaidacea from the Mediterranean basin, the Aegean Sea, due to its special relief (i.e. a very large number of islands of different size and origin), consists of a large diversity of biocoenoses with ecological niches that can become "deposits" for species and even genera still unknown. Actually, there are just a few studies that have been carried out along the coasts of the Aegean Sea recording tanaidacean taxa (Bakır et al. 2014, Bakır and Çevirgen 2010, Kocataş et al. 2004). As mentioned above, Stefanidou (1996) is the one who studied the Tanaidacea of the continental shelf of the North Aegean Sea and the only one who largely contributed to the Greek records of this taxonomic group. Until now, only scattered distribution data of already recorded tanaidacean taxa have been published as part of crustacean assemblages in Greek Seas (Baxevanis and Chintiroglou 2000, Antoniadou 2004, Chintiroglou et al. 2004, Koulouri et al. 2013). There are also a few studies referring to tanaidacean taxa associated with sponges and corals (Koukouras et al. 1985, Koukouras et al. 1996, Koukouras et al. 1998). Furthermore, it is worth mentioning that, recently, the tanaids *Chondrochelia savignyi* and *Paradoxapseudes intermedius*, associated with sponges, have been recorded in submarine caves of Lesvos Island (North Aegean Sea) by Gerovasileiou et al. (2016).

Surprisingly, the Mediterranean Sea is thought to be a well-studied area (Blazewicz-Paszkowycz et al. 2012), as recent investigations of the eastern Mediterranean fauna found high densities of previously undescribed tanaidacean species (Bamber et al. 2009). Until now and to our knowledge, 69 tanaidacean taxa (Suppl. material 1) have been found in the Mediterranean Sea (Guţu 2002, Bamber et al. 2009, Anderson 2013, Costello et al. 2020, WoRMS Editorial Board 2020), including the Erythrean *Cristapseudes omercooperi* present in the eastern Mediterranean which is most likely to be associated with transport via shipping through the Suez Canal (Bamber et al. 2009). As a very few species are known from deep waters in the Mediterranean Sea (Kudinova-Pasternak 1982), the large majority of deep-sea tanaidaceans have yet to be discovered and described (Blazewicz-Paszkowycz et al. 2012).

Considering the low mobility of tanaids (minimal inherent dispersive ability), wide distribution of several species could be the result of passive dispersion or aggregates of sibling species (Blazewicz-Paszkowycz et al. 2012). Indeed, there are tanaid species in the list which appear to be widely distributed, probably due to passive dispersion. The presence of *Tanais dulongii* has been attributed to possible transport on ships’ hulls (Blazewicz-Paszkowycz et al. 2012). One species of the family Tanaididae, *Hexapleomera robusta*, has been found living in relatively high densities in its tubes on the carapaces of marine turtles (Blazewicz-Paszkowycz et al. 2012). This behaviour has the potential for a wide distribution of commensal tanaidaceans until they become detached from their host. The species *Apseudopsis acutifrons*, included in this study, also appears to be widely distributed as it has been found, not only in the Aegean and the Levantine Seas (Stefanidou 1996, Bamber et al. 2009), which are part of the eastern Mediterranean basin, but also in the western Mediterranean Sea (Guţu 2002). Furthermore, species, such as *Apseudes holthuisi* and *Apseudopsis mediterraneus*, have been reported from the Aegean and the Levantine Seas (Stefanidou 1996, Bamber et al. 2009), while *Apseudopsis ostroumovi* has been reported from the Levantine and the Black Seas (Guţu 2002, Bamber et al. 2009). On the other hand, the species *Apseudes robustus*, *Apseudopsis latreillii*, *Chondrochelia savignyi*, *Leptochelia mergellinae*, *Paradoxapseudes intermedius*, *Pseudoleptochelia anomala* and *Pseudoparatanais batei* have been identified by different researchers in different areas of the Aegean Sea (Koukouras et al. 1985, Koukouras et al. 1996, Koukouras et al. 1998, Antoniadou 2004, Chintiroglou et al. 2004, Bakır and Çevirgen 2010, Bakır et al. 2014). Presence of new species in material from Greece, however, is probable as several widely-distributed species could also be aggregates of sibling species. Therefore, re-examination of existing material and further research on tanaid fauna in Greek Seas is necessary, especially by using current molecular techniques.

Tanaidaceans are amongst the smallest benthic macro-invertebrates. Therefore, the use of coarse meshes in gears or in sample treatment have probably missed many of them until now (Blazewicz-Paszkowycz et al. 2012). The use of 500 mesh sieves for analysis of soft-sediment benthos is entirely appropriate at the community level, yet inevitably loses a significant proportion of the tanaidacean fauna, particularly interstitial species (Bamber et al. 2009, Blazewicz-Paszkowycz et al. 2012). Moreover, for the study of crustacean/hyperbenthic assemblages, including tanaidaceans, specially designed sledges have been constructed, modified and used on soft bottoms over the last 40 years (Mees and Jones 1997, Eleftheriou 2013). However, most of these hyperbenthic sledges do not sample the supernatant layer above the seabed in order to avoid contamination of the samples by sediment (Mees and Jones 1997, Eleftheriou 2013). Nevertheless, Koulouri et al. (2013) applied a modified hyperbenthic sledge specifically designed to re-suspend the sediment surface and results revealed that this demersal macrofauna, including tanaidaceans, is concentrated in the lowermost layer of the water column, just above the seabed surface, in the oligotrophic environment of the Mediterranean Sea. Therefore, apart from the taxonomists who have to be encouraged to deal with inherent identification difficulties when characterising tanaids, effective sampling efforts should be intensified and sample treatment should be taken into account.

## Supplementary Material

CB7400DA-C049-571C-8DB3-B3BAD4FA3EE2 10.3897/BDJ.8.e47184.suppl1 Supplementary material 1 Tanaidacea of Greece and the Mediterranean Sea Data type Checklist Brief description Checklists of the tanaidacean fauna of Greece and the Mediterranean Sea File: oo_383253.xlsx https://binary.pensoft.net/file/383253 Panayota Koulouri, Vasilis Gerovasileiou, Nicolas Bailly, Costas Dounas

XML Treatment for Tanaidacea

XML Treatment for Apseudidae

XML Treatment for Apseudes africanus

XML Treatment for Apseudes holthuisi

XML Treatment for Apseudes orientalis

XML Treatment for Apseudes robustus

XML Treatment for Apseudes talpa

XML Treatment for Apseudopsis acutifrons

XML Treatment for Apseudopsis elisae

XML Treatment for Apseudopsis latreillii

XML Treatment for Apseudopsis mediterraneus

XML Treatment for Paradoxapseudes intermedius

XML Treatment for Leptocheliidae

XML Treatment for Chondrochelia savignyi

XML Treatment for Leptochelia inermis

XML Treatment for Leptochelia mergellinae

XML Treatment for Pseudoleptochelia anomala

XML Treatment for Leptognathiidae

XML Treatment for Leptognathia breviremis

XML Treatment for Pseudoparatanais batei

XML Treatment for Parapseudidae

XML Treatment for Parapseudes latifrons

XML Treatment for Tanaididae

XML Treatment for Hexapleomera robusta

XML Treatment for Tanais dulongii

XML Treatment for Tanais grimaldii
